# Residual effect of commonly used insecticides on key predatory mites released for biocontrol in strawberry

**DOI:** 10.1093/jee/toae220

**Published:** 2024-10-10

**Authors:** Allan Busuulwa, Simon S Riley, Alexandra M Revynthi, Oscar E Liburd, Sriyanka Lahiri

**Affiliations:** Entomology and Nematology Department, Gulf Coast Research and Education Center, University of Florida, Wimauma, FL, USA; Statistical Consulting Unit, Institute of Food and Agricultural Sciences, University of Florida, Gainesville, FL, USA; Entomology and Nematology Department, Tropical Research and Education Center, University of Florida, Homestead, FL, USA; Entomology and Nematology Department, University of Florida, Gainesville, FL, USA; Entomology and Nematology Department, Gulf Coast Research and Education Center, University of Florida, Wimauma, FL, USA

**Keywords:** *Scirtothrips dorsalis*, *Amblyseius swirskii*, *Neoseiulus cucumeris*, *Neoseiulus californicus*, nontarget effects

## Abstract

Florida is the second largest producer of strawberries in the United States. However, the production system faces numerous challenges, especially *Scirtothrips dorsalis* Hood (Thysanoptera: Thripidae) infestations. Management of this pest involves applying insecticides and use of predatory mites, particularly *Amblyseius swirskii* Athias-Henriot, *Neoseiulus cucumeris* Oudemans, and *Neoseiulus californicus* McGregor (Mesostigmata: Phytoseiidae). Strawberry growers in Florida are concerned about the compatibility of the commercial formulations of insecticides used in strawberry pest management with predatory mites. This study assessed the residual effect of commercial insecticides used in strawberry production on the survival, feeding, and oviposition of the 3 predators. Using Munger cells, predators were exposed to commercial formulations of spinetoram, cyantraniliprole, azadirachtin + pyrethrin, *Beauveria bassiana*, *Cordyceps javanica*, capsicum, garlic, and canola oil extracts, and water control. There was a gradual decline in the survival and feeding of predatory mites when exposed to all insecticides. Spinetoram had the highest impact on the survival and feeding of all predators compared to other insecticides, while *C*. *javanica* had the lowest impact. Cyantraniliprole and azadirachtin + pyrethrin significantly reduced predator survival after 72 h of exposure, whereas capsicum, garlic, and canola oil extracts caused a similar reduction after 96 h. All predators consumed low proportions of *S. dorsalis* across all treatments. Oviposition was low in all treatments, with no discernable variation among treatments. These results highlight the potential of using entomopathogenic fungi in conjunction with *A. swirskii*, *N. cucumeris*, and *N. californicus* for the management of *S. dorsalis* and *T. urticae* in strawberries.

## Graphical Abstract

**Graphical Abstract:**
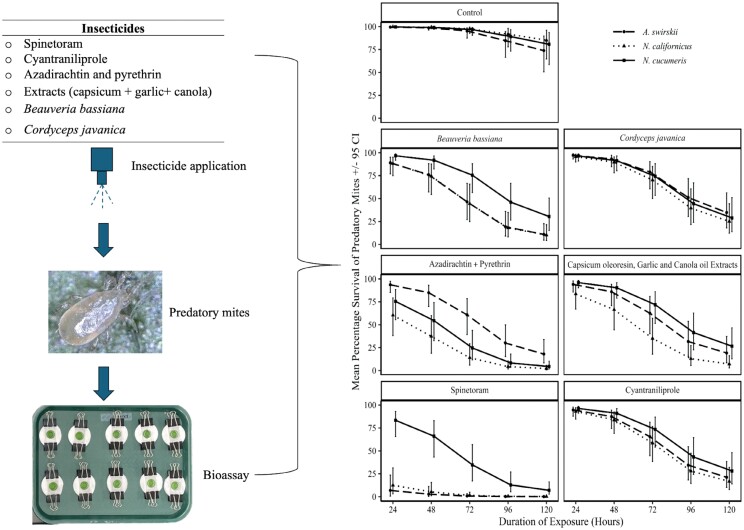

## Introduction

Strawberry *Fragaria* × *ananassa* (Rosaceae) production significantly contributes to the US economy, especially in California and Florida, the top-producing states. The state of Florida is the second-largest producer and the overall top producer of winter strawberries (Guan et al. 2016, Huang et al. 2022). Similar to other agricultural sectors, strawberry production faces significant challenges, particularly from a variety of arthropod pests. In Florida, the primary strawberry pest complex includes various thrips species such as *Scirtothrips dorsalis* Hood, *Frankliniella occidentalis* Pergande, and *Frankliniella bispinosa* Morgan (Thysanoptera: Thripidae), of which the *S. dorsalis* is the most severe pest (Lahiri and Panthi 2020, Panthi and Renkema 2020, Panthi et al. 2021). The pest complex also contains a wide range of phytophagous mite species, such as *Tetranychus urticae* Koch (Trombidiformes: Tetranychidae), *Polyphagotarsonemus latus* Banks, and *Phytonemus pallidus* Banks (Trombidiformes: Tarsonemidae), with *T. urticae* being the most prevalent mite pest (Akyazi and Liburd 2019, Lahiri et al. 2022, 2024, Montemayor et al. 2023, Busuulwa et al. 2024). In some strawberry fields, it is possible to find co-occurring infestations of *S. dorsalis* and *T. urticae* (Lahiri et al. 2024).

To manage *S. dorsalis* and *T. urticae*, the majority of strawberry growers in Florida rely on insecticide applications (Lahiri and Panthi 2020, Panthi and Renkema 2020, Gireesh et al. 2022, Lahiri et al. 2022, Lahiri 2023). Some of the most commonly used insecticides in strawberry production include broad-spectrum reduced-risk synthetic insecticides such as spinetoram and cyantraniliprole. Plant-derived insecticides such as capsicum oleoresin, garlic oil, and canola oil extracts, and azadirachtin + pyrethrin are also widely used. Additionally, entomopathogenic fungi especially *Beauveria bassiana* strain GHA and *Cordyceps javanica* (formally known as *Isaria fumosorosea*) are used by some growers during the strawberry season. However, due to increasing concerns about the development of resistance to some of the reduced-risk insecticides (Kaur et al. 2023), the augmentative release of biological control agents, particularly phytoseiid mites, has become a common practice among growers (Lahiri et al. 2022, 2024, Lahiri 2023).

Currently, the most commonly used predatory mites include *Neoseiulus cucumeris* Oudemans, *Neoseiulus californicus* McGregor, *Amblyseius swirskii* Athias-Henriot, and *Phytoseiulus persimilis* Athias-Henriot (Mesostigmata: Phytoseiidae). *Neoseiulus cucumeris*, *N. californicus*, and *A. swirskii* are generalist predators (McMurtry and Croft 1997, 2003, McMurtry et al. 2013) that can feed on a variety of prey species in addition to pollen. In contrast, *P. persimilis* is a specialist predator of spider mites. *Amblyseius swirskii* and *N. cucumeris* have been used to control important agricultural pests such as *Bemisia tabaci* (Gennadius) (Hemiptera: Aleyrodidae) (Nomikou et al. 2002, Li et al. 2017), and thrips (Zilahi-Balogh et al. 2007, Arthurs et al. 2009, Kakkar et al. 2016, Lahiri and Yambisa 2021, Schoeller et al. 2022) while *N. californicus* has been extensively used to manage *T. urticae* (Rhodes et al. 2006, Gotoh et al. 2007, Rahmani et al. 2016). The ability of *A. swirskii*, *N. cucumeris*, and *N. californicus* to feed on various mite species has significantly enhanced their mass-rearing and facilitated their commercialization on a large scale (Massaro et al. 2016). Since these predators can also survive on pollen (McMurtry et al. 2013), they are able to maintain stable populations in the field even when pest populations are low, thereby providing constant pest suppression. As a result, these qualities have made them the preferred augmentative biocontrol agent for *S. dorsalis* management in strawberries.

However, it is still common to encounter strawberry growers applying insecticides and releasing predatory mites concurrently in the same field, a practice done as part of their integrated pest management (IPM) strategy to effectively suppress *S. dorsalis* populations. Several laboratory studies have shown that most of the insecticides used in various cropping systems negatively affect many species of phytoseiid predatory mites by reducing their survival rate, predation, and in some instances oviposition. For example, imidacloprid, fenpyroximate, and lambda-cyhalothrin were found to be extremely toxic to *A. swirskii*, *P. persimilis* and *Amblyseius andersoni* (Chant) (Fiedler and Sosnowska 2014).

Fenazaquin, an acaricide with both contact and ovicidal activity, was reported to decrease the developmental time of *A. swirskii*, and that of its successive generations, while acetamiprid caused a significant decline in survival and fecundity of the predatory mite (Shahbaz et al. 2019). Similarly, high mortality of *A. swirskii* was observed when it was exposed to fenpyroximate (Fiedler and Sosnowska 2014). Although a combination of fenpyroximate and thiacloprid at their reduced rate was reported to be moderately toxic to *A. swirskii* (Ghasemzadeh and Qureshi 2018), applications of thiacloprid alone significantly reduced the survival and oviposition of the predatory mite.

Exposure of *A. swirskii* to abamectin and pyridaben was reported to result into high mortality rates for all developmental stages of the predator, with the highest mortality occurring in adult females (Döker and Kazak 2019). Similarly, exposure of *Iphiseius degenerans* (Berlese) (Mesostigmata: Phytoseiidae) to spinetoram resulted in high mortality of the predator (Döker et al. 2015). High acetamiprid concentrations were reported to heavily reduce feeding, oviposition, and survival of *N. cucumeris* (Cheng et al. 2018). Azadirachtin, a biorational insecticide although reported to be nontoxic to *Stratiolaelaps scimitus* (Womersley) (Mesostigmata: Laelapidae) was found to be moderately toxic to *Galendromus occidentalis* (Nesbitt) (Mesostigmata: Phytoseiidae) (Yanar 2019), while spinetoram applications were found to cause high mortality of the predatory mite (Beers and Schmidt 2014).

Considering that strawberry growers in Florida perform weekly insecticide and fungicide applications in their fields alongside the release of predatory mites, it is essential to examine the effects of commonly used insecticides to assess their compatibility with these beneficial predators. Such research would offer the foundational information necessary for developing IPM programs that allow for the incorporation of predatory mites. Thus, the main aim of this study was to determine the compatibility of commonly used conventional and biorational pesticides with predatory mites by comparing their effect on the feeding, oviposition, and survival of *A. swirskii*, *N. cucumeris*, and *N. californicus*.

## Materials and Methods

### Predatory Mite Rearing

*Amblyseius swirskii*, *N. cucumeris*, and *N. californicus*, used in the experiment, were initially sourced from Arbico Organics (Tucson, AZ, USA) and then placed in laboratory culture. To start the laboratory colonies used in the bioassays, 200 gravid females of each predator species were transferred onto separate rearing arenas using a fine paint brush. Gravid females were identified by their distinctly enlarged, round-shaped opisthosomas.

The rearing arenas used in this experiment were similar to those described by Helle and Sabelis (1985). Each arena comprised a plastic dish pan (35.6 × 29 × 12 cm, Greenbrier International, Inc., USA) half-filled with distilled water. Large multipurpose sponges (19 × 14 × 2.5 cm, QEP, Boca Raton, FL, USA) were placed in the pans on which a black polystyrene flexible plastic board (12 × 8 cm, MEGA Format, Brooklyn, NY, USA) was placed. The edges of the plastic boards were lined with moist, nonsterile cotton (Fisher Scientific, NJ, USA) to prevent the predators from escaping.

To facilitate oviposition, triangular structures were created from small plastic sheets, and cotton fibers were then adhered to the underside of these structures, which were then placed on the arena. These structures provided suitable spots for the predators to lay their eggs. Once prepared, the arenas were transferred to a growth chamber, maintained at 25 ± 1 °C, 70 ± 5% RH, and 14:10 h L:D. To sustain the established colonies, a mixture of ~300–400 first and second-instar larvae of *S. dorsalis* were provided as a food source every 48 h, by gently brushing them onto the arena using a paintbrush. Both first and second instar larvae of *S. dorsalis* were provided because of the predators’ capability to feed on both developmental stages (Arthurs et al. 2009). *Scirtothrips dorsalis* larvae used as a food source were obtained from laboratory colonies raised on cotton plants in a growth room, where the conditions were kept at 25 ± 1 °C, 65 ± 5% RH, and 14:10 h L:D.

To obtain predators of the same age, 120 gravid female predatory mites were randomly selected from the primary colony and placed into individual rearing arenas for egg-laying. After a 24-h oviposition period, the females were removed, and the arenas with the eggs were kept in a growth chamber at 26 ± 1 °C, 70 ± 5% RH, and 14:10 h L:D to ensure optimal conditions for the eggs to hatch. Upon hatching, the predatory mite nymphs were provided with first and second instar larvae of *S. dorsalis* by brushing approximately 200 larvae onto each rearing arena. This procedure was repeated at 48-h intervals, culminating when the predatory mites matured into adults and commenced oviposition, which occured 8 days after hatching. This predatory mite generation was then used for all following experiments.

### Insecticides

Six insecticides commonly used in *S. dorsalis* management in strawberry production in Florida were tested (Table 1). The insecticides were categorized into 2 broad groups: reduced-risk insecticides (spinetoram and cyantraniliprole) and biopesticides (*Beauveria bassiana*, *Cordyceps javanica*, azadirachtin + pyrethrin and capsicum oleoresin, garlic oil, and canola oil extracts (Leahy et al. 2014). The biopesticides were further divided into 2 categories: the entomopathogenic fungi (*Beauveria bassiana* and *Cordyceps javanica*), and plant extracts (azadirachtin + pyrethrin and capsicum oleoresin, garlic oil, and canola oil extracts).

**Table 1.** List of insecticides tested on predatory mites, including their trade names, active ingredients, and the maximum recommended application rates for strawberries specified by the manufacturers

Trade name	Active ingredient (AI) and percentage composition	Chemical class	Insecticide type	Application rate
Radiant SC	Spinetoram (11.7%)	Spinosyns	Reduced risk	0.88 L/ha
Exirel	Cyantraniliprole (10.2%)	Diamides	Reduced risk	1.5 L/ha
Azera	Azadirachtin (1.20%) and Pyrethrin (1.40%)	Pyrethrin	Plant extract	4.1 L/ha
Captiva Prime	Capsicum oleoresin (7.60%), garlic oil (23.40%), and canola oil (55.00%) extracts	Botanical essence	Plant extract	2.4 L/ha
Mycotrol ESO	*Beauveria bassiana* strain GHA (11.30%)	Fungal agents	Entomopathogenic fungi	4.7 L/ha
PFR-97 20% WDG	*Cordyceps javanica* formally *Isaria fumosorosea* Apopka Strain 97 (20.0%)	Fungal agents	Entomopathogenic fungi	2.24 g/ha

### Strawberry Plants

“Brilliance” cultivar strawberry transplants were grown in plastic pots inside an insect-rearing cage. The cage was kept in a growth chamber with the temperature set at 25 ± 1 °C, relative humidity at 65 ± 5%, and a light-dark cycle of 14:10 L:D. Plants were watered and fertilized as needed. The plants were grown for 6 weeks before being used in the experiments.

### Residual Contact Toxicity of Insecticides to Predatory Mite Adult Females

Leaf discs measuring 12 mm in diameter were cut from *S. dorsalis*-free plants from the growth chamber. The leaf discs were then immersed for 10 s in an insecticide solution that had been prepared using the manufacturer’s maximum strawberry recommended application rate for the management of *S. dorsalis* (Table 1). A control treatment, created by dipping the leaf discs in distilled water for 10 s, was included in the experiment. After the dipping process, the treated leaf discs were left to air dry for 1.5 h before being used in the experiment. Experimental arenas used were similar to those used by Busuulwa et al. (2024), which were closely modeled after those described by Helle and Sabelis (1985) and Argolo et al. (2020).

In brief, the arenas were constructed using 2 transparent acrylic glass plates, each measuring 75 mm by 26 mm. One of the glass plates had a central circular hole with a diameter of 12.7 mm, designed to fit within the outline of the leaf disc used in the experiment. The second glass plate, identical in size, served as the base of the setup. A layer of moist cotton was placed on this base plate, on top of which a leaf disc with the abaxial surface facing downward was placed. The glass plate with the hole was then carefully placed on top of the leaf disc, creating a sandwich-like structure.

In each arena, a single 10-day-old female predator was randomly selected from the age-synchronized colony and carefully placed onto the treated strawberry leaf disc. To serve as a food source, 10 *S. dorsalis* larvae (first and second instar) were introduced into the same arena with the predatory mite. Each treatment (insecticides and the control) consisted of 10 replicates. After the experimental setup, the arenas were transferred to a growth chamber maintained at 25 ± 1 °C, 65 ± 5% RH, and 14:10 h L:D.

*Scirtothrips dorsalis* larvae were added to the arenas every 24 h to replenish those consumed by the predatory mites. Data on the number of predatory mites alive (survival), the number of *S. dorsalis* larvae consumed (feeding), and the total number of egg produced by the predators (oviposition rate) was recorded at 24-h intervals for 120 h. *Scirtothrips dorsal* larvae that had been fed on by the predators were easily distinguishable from those that had died of other causes given that the former were desiccated. During the course of the experiment, eggs laid by the predators were not removed from the experimental arena to avoid disturbing the adult females and to prevent the potential escape of *S. dorsalis* larval prey. As a result, the number of eggs laid during each period was determined by subtracting the egg count from the previous day. Nevertheless, the viability of the eggs was not assessed, as it was beyond the scope of this study. The whole experimental setup was conducted twice to ensure consistency and reliability of the results obtained.

### Statistical Analysis

The Bayesian framework (Ellison 2004) was utilized to test our hypothesis that both conventional and biopesticides possess some negative effects on predatory mites. This approach was chosen primarily for the fact that it allows the use of regularizing priors, which can improve parameter identifiability and generate more robust estimates compared with maximum-likelihood based methods (McElreath 2020). Overall, the experiment was structured as a completely randomized design with a split-plot restriction on randomization, wherein there were 2 replicates of the main plot factor (predatory mite species) and 10 replicates for each insecticide and control treatment (subplot factor). In addition, the study involved repeated measures on each individual leaf disc taken at 5 time points. Separate analyses, described below, were conducted for predatory mite survival, feeding, and oviposition. For each model, we executed 8 chains and performed 25,000 iterations, with 20,000 of those iterations designated as warm-up iterations. All analyses were conducted in R version 4.0.3 (R Core Team 2024) and Stan (version 2.30) (Bürkner 2021, Stan Development Team 2022, Guo Jiqiang et al. 2024).

Predatory mite survival was modeled using ordinal logistic regression, with mite species treated as a fixed effect while the effects of insecticides, the insecticide-by-species interaction, and the main plot experimental units (“2 trials,” the whole experimental repeated twice), were treated as random effects. The former 2 random effects were treated as such to generate partially pooled estimates (Hobbs and Hooten 2015), which were especially desirable because for some combinations of predator and insecticide, no predators survived to the first observation period. The proportion of *S. dorsalis* consumed by the predators throughout the 120-h period of observation was assumed to be binomially distributed, and thus predatory mite feeding was modeled using a generalized linear mixed-effects model (GLMM) (Bolker et al. 2009), with the specification of fixed and random effects the same as in the analysis of mite survival. The proportion of *S. dorsalis* larvae consumed by the predators was calculated as number of *S. dorsalis* consumed every 24 h divided by the total number of *S. dorsalis* larvae provided (10 larvae). Given that predators consistently consumed low proportions of *S. dorsalis* over the entire observation period, a regression model was fitted, slope calculated, and comparisons between the slopes made using 120-h as the cutoff point.

Predatory mite oviposition, recorded as the daily number of eggs produced (oviposition rate) was also modeled using a GLMM, but with the assumption that egg production had a Poisson distribution and with a First-order Autoregressive Covariance Structure (AR1) among measures taken from the same leaf disc over time. A Poisson distribution was chosen in this case because using a negative binomial and an autoregressive correlation structure rendered the model overparameterized and unidentifiable. The predatory mite species were treated as fixed effects. In all cases, fixed effects were given weakly informative normal priors with mean zero. Random effect standard deviations were given weakly informative half-Cauchy priors, and the cut points in the ordinal logistic regression were given induced Dirichlet priors with concentration parameters equal to one (for details, see Betancourt (2019)).

After fitting the models, preplanned orthogonal contrasts were used to estimate, compare, and test the effects of different groups of insecticides on the survival, feeding, and oviposition of the different predatory mites, as shown in (Table 2). Such contrasts provide more focused and meaningful comparisons than those achieved via all pairwise comparisons (Saville and Graham 2012). Therefore, preplanned orthogonal contrast that leveraged relationships between the insecticides and predatory mites were developed. These comparisons assessed the probability of predatory mites surviving, the proportion of prey consumed, and the rate of oviposition for 120 h under treatment, considering the demonstrated residual activity of the insecticides used, especially spinetoram, which lasts between 3 and 7 days (Shimokawatoko et al. 2012, Depalo et al. 2016). To detect significant differences between contrasts, a comparison of posterior distributions was performed. This was done by computing the product of the Lower and Upper Credible Interval (LCL/UCL) and determining whether it overlaps with zero (LCL*UCL > 0).

**Table 2.** Preplanned orthogonal contrasts designed to compare the percentage of predatory mites alive (survival), number of *S. dorsalis* consumed by the predatory mites (feeding), and daily number of eggs laid by the predatory mites (oviposition rate) after exposure to different groups of insecticide treatments

Contrast	Name	Description
C1	Control—Insecticide	Predatory mites on insecticide-treated leaf discs vs. those in the control treatment
C2	Biopesticide—Reduced-risk insecticide	Predatory mites on leaf discs treated with a reduced-risk insecticide (cyantraniliprole or spinetoram) vs. those on leaf discs treated with a biopesticide (azadirachtin + pyrethrin, capsicum canola, and garlic oil extracts, *Beauveria bassiana*, or *Cordyceps javanica*)
C3	Plant Extract—Entomopathogenic insecticide	Predatory mites on leaf discs treated with an entomopathogenic insecticide (*Beauveria bassiana* or *Cordyceps javanica*) vs. those on leaf discs treated with a plant extract-based insecticide (azadirachtin + pyrethrin or capsicum canola and garlic oil extracts)
C4	Spinetoram—Cyantraniliprole (between reduced-risk insecticides)	Predatory mites on leaf discs treated with cyantraniliprole vs. those on leaf discs treated with spinetoram
C5	*Beauveria bassiana*—*Cordyceps javanica*. (between entomopathogenic insecticide)	Predatory mites on leaf discs treated with *Cordyceps javanica* vs. those on leaf discs treated with *Beauveria bassiana*
C6	Azadirachtin + Pyrethrin—Capsicum, garlic, and canola oil extracts (between plant extracts insecticides)	Predatory mites on leaf discs treated with capsicum canola and garlic oil extracts vs. those on leaf discs treated with azadirachtin + pyrethrin

The “Contrast” column contains the abbreviation /code for the contrast. The “Name” column lists the conditions being contrasted, with the first stated category regarded as the first condition and the second category as the second condition. For example, for C1, “Control” is Condition 1, and “Insecticide” is Condition 2; similarly, for C2, “Biopesticide” is Condition 1, and “Reduced risk insecticide” is Condition 2. The “Description” column provides details of the contrast.

## Results

### Overall Survival

After 120 h, predatory mites exposed to reduced-risk insecticides had the lowest survival, 12.5%, with a 95% credible interval (CI) of 8.4%–18.0% compared to those exposed to the 2 types of biopesticides (18.5%, CI: 13.7%–24.3%). Predators exposed to plant extracts had lower survival (13.1%, CI: 8.7%–19.0%) than those exposed to entomopathogenic fungal insecticides (23.7%, CI: 17.3%–31.9%). The highest predatory mite survival was observed in the control group at 81.6% (CI: 69.6%–90.5%) compared to all other treatments (16.5%, CI: 12.5%–21.3%). However, the analysis also revealed significant variation within each insecticide type (Table 3).

**Table 3.** Percentage of predatory mites alive after 120 h of exposure to different groups of insecticide treatments. Comparisons are based on the preplanned contrasts

Contrast	Predatory mite survival marginal means (%)		Difference between survival marginal means (*Δ*μ)	*Δ*μ LCL	*Δ*μ UCL	UCL*LCL > 0
C1	79.17	16.73**(%)**	62.44	52.8	74.2	*
C2	18.78	12.58	6.2	0.7	11.3	*
C3	13.41	23.99	−10.58	17.9	−3.6	*
C4	2.47	22.27	−19.8	29.6	−12.1	*
C5	17.61	30.16	−12.55	24.7	−1.1	*
C6	8.44	18.10	−9.66	18.6	−0.22	*

The “Predatory mite survival marginal means (%)” column includes 2 subcolumns that show the mean percentage survival of predatory mites across 3 species for the 2 conditions being compared. The conditions are listed in the same order as described in Table 2. The differences in marginal means (Δ μ) were calculated by subtracting the mean of condition 1 from that of condition 2 in each contrast. Positive values indicate higher percentage survival of predatory mites for condition 1 of the contrast, while negative values indicate higher survival for condition 2. The “LCL” and “UCL” columns show the lower and upper credible intervals of *Δ*μ, respectively. Asterisks (*) indicate significant differences between the contrast comparisons. Significance was computed by establishing whether the product of UCL and LCL overlap with zero.

On average, among reduced-risk insecticides, spinetoram had the lowest predatory mite survival (2.5% CI: 1.0%–5.4%) compared to cyantraniliprole (22.3% CI 14.7%–32.7%) after 120 h of exposure. Between the plant extract group, azadirachtin + pyrethrin had the lowest predator survival (8.2%, CI: 4.5%–13.8%) compared to capsicum oleoresin, garlic, and canola oil extracts (18.0%, CI: 11.1%–27.1%). Upon comparing the entomopathogenic fungal insecticides, *B. bassiana* had lower predator survival (17.5%, CI: 11.1%–26.3%) compared to *C. javanica* (18.0% CI: 11.1%–27.1%).

### Survival by Predator Species

**Fig. 1.**
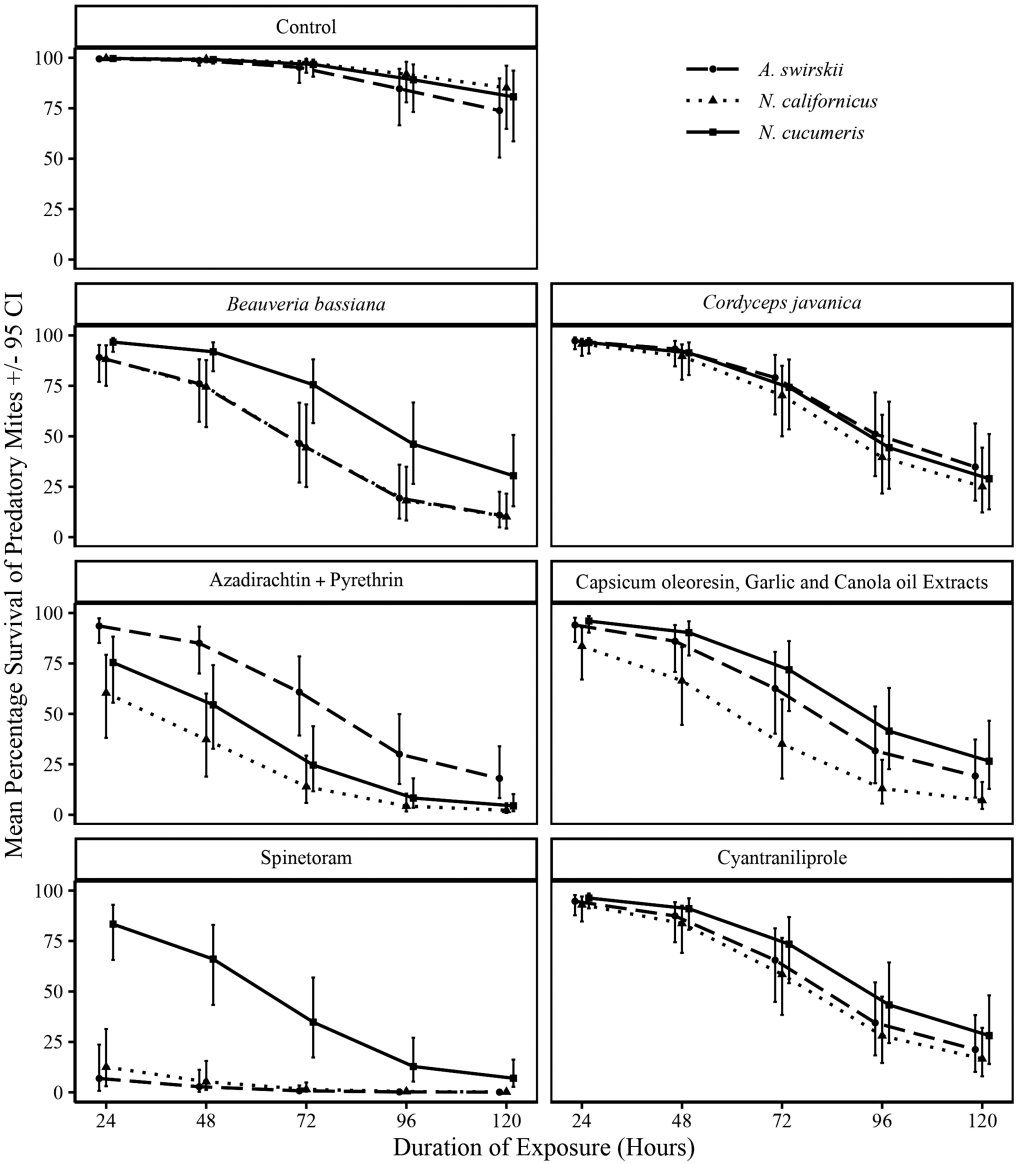
Percentage of *A. swirskii*, *N. californicus*, and *N. cucumeris* alive at various time points following exposure to insecticide treatments.

The impact of insecticides on the survival of predators varied across predatory mite species. In all treatments, we observed a decrease in predator survival with prolonged exposure to insecticides (Fig. 1). When exposed to spinetoram, *A. swirskii* and *N. californicus* had very low survival (6.8%, CI: 0.7%–20.4% and 12.5%, CI: 3.1%–31.4%, respectively), compared to *N. cucumeris* (83.4%, CI: 65.6%–92.9%). However, there was a substantial decline in *N. cucumeris* survival by 72 h of exposure (34.8%, CI: 17.3%–56.8%).

When exposed to azadirachtin + pyrethrin, *A. swirskii* consistently had higher survival even at 72 h (60.8%, CI: 39.3%–78.5%) compared to *N. californicus* (13.9%, CI: 5.9%–29.3%) and *N. cucumeris* (24.6%, CI: 11.6%–43.9%). *Neoseiulus cucumeris* exhibited higher survival in the *B. bassiana* treatment compared to *A. swirskii* and *N. californicus*, especially after 72 h (*N. cucumeris*: 75.5%, CI: 56.6%–88.1%; *A. swirskii*: 46.4%, CI: 27.1%–66.6%; *N. californicus*: 44.4%, CI: 24.9%–65.8%), and 120 h of exposure (*N. cucumeris*: 30.4%, CI: 15.3%–50.1%; *A. swirskii*: 10.9%, CI: 4.8%–22.5%; *N. californicus*: 10.1%, CI: 4.3%–21.5%). However, there were no differences in predatory mite survival when exposed to *C. javanica*, cyantraniliprole, capsicum oleoresin, garlic, and canola oil extracts.

### Feeding (Proportion of *S. dorsalis* Consumed)

The highest proportion of prey consumed averaged across all 3 predators was observed in the control (0.30, CI: 0.26–0.382). Predators exposed to entomopathogenic had higher proportions of prey consumed compared to those exposed to plant extracts (0.20, CI: 0.17–0.27). Reduced-risk insecticides had the lowest proportion of *S. dorsalis* consumed (0.17, CI: 0.14–0.21) compared to all other treatments (Table 4).

**Table 4.** Pooled proportion of *S. dorsalis* larvae (prey) consumed by the predatory mites after exposure to different insecticide treatments. Comparisons are based on the preplanned contrasts

Contrast	Proportion of prey consumed		Difference between proportion of prey consumed (*Δ*μ)	*Δ*μ LCL	*Δ*μ UCL	UCL*LCL > 0
C1	0.30	0.20**(%)**	0.097	0.057	0.138	*
C2	0.22	0.17	0.054	0.027	0.085	*
C3	0.20	0.24	−0.039	−0.073	−0.006	*
C4	0.04	0.28	−0.238	−0.301	−0.189	*
C5	0.19	0.29	−0.092	−0.140	−0.044	*
C6	0.17	0.22	−0.054	−0.102	−0.011	*

The “Proportion of prey consumed” column contains 2 subcolumns that show the proportion (out of 10) of *S. dorsalis* larvae consumed averaged across the 3 predatory mite species for the 2 conditions being contrasted. The conditions are listed in the same order as described in Table 2. Differences in the proportion of prey consumed (*Δ*μ) were calculated by subtracting the mean of condition 1 from that of condition 2 in each contrast. Positive values indicate a higher proportion of prey consumed for condition 1, while negative values indicate a higher proportion of prey consumed for condition 2. The “LCL” and “UCL” columns show the lower and upper credible intervals of Δμ, respectively. Asterisks (*) indicate significant differences between the contrast comparisons, determined by whether the product of UCL and LCL overlap with zero.

**Fig. 2.**
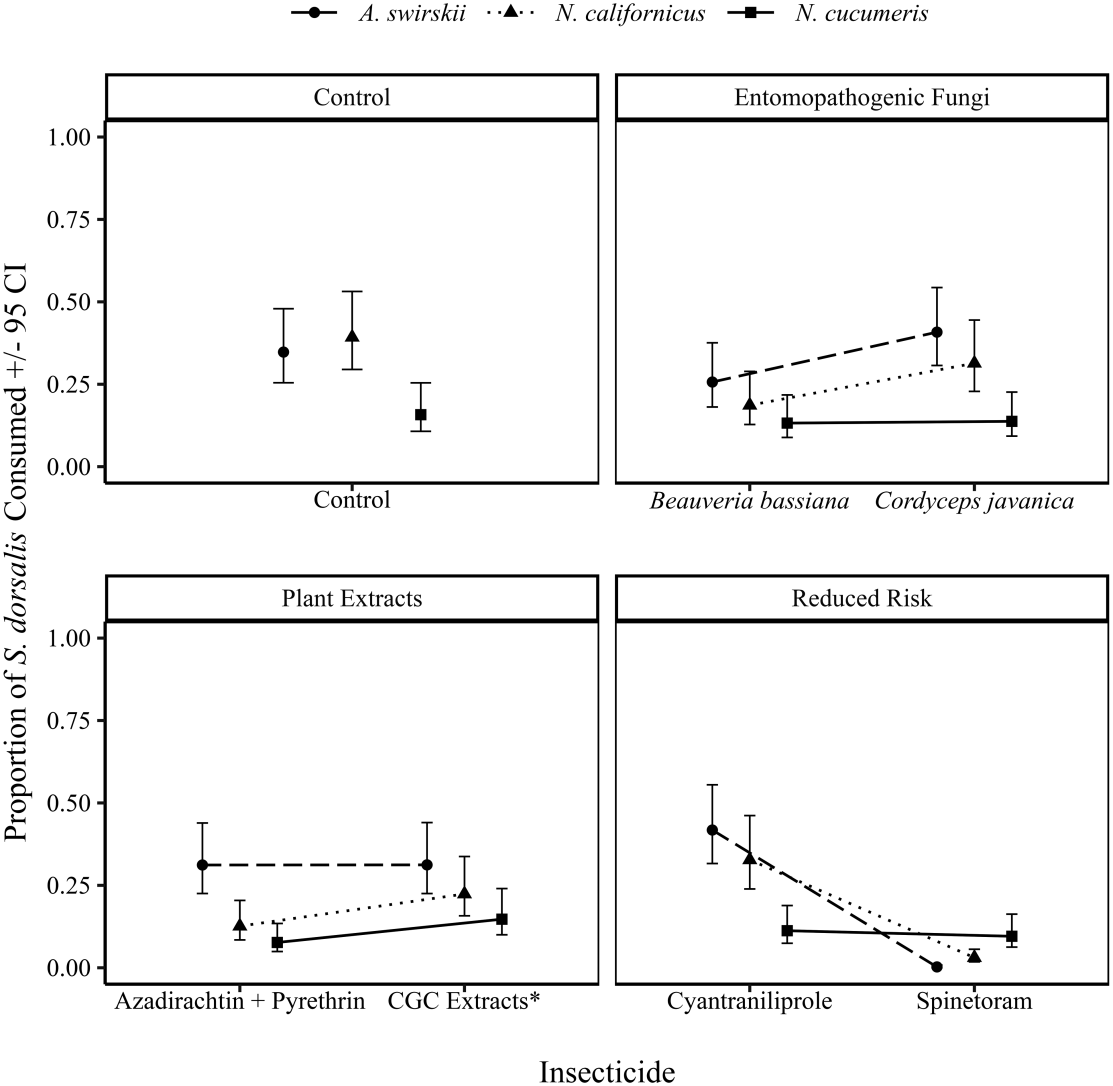
The average proportion of *S. dorsalis* consumed by *A. swirskii*, *N. californicus*, and *N. cucumeris* throughout the experiment *CGC extracts represents capsicum, garlic, and canola oil extracts.

The results also indicated that by 120 h, within the control treatment, *N. californicus* and *A. swirskii* had the highest proportions of prey consumed (*N. californicus*: 0.40, CI: 0.28–0.52; *A. swirskii*: 0.35, CI: 0.25–0.48) compared to *N. cucumeris* (0.16 CI: 0.10–0.26). Within the entomopathogenic group, *A. swirskii* had a higher proportion of prey consumption (0.25, CI: 0.17–0.37) in comparison *to N. californicus* (0.18, CI: 0.12–0.29) and *N. cucumeris* (0.13, CI: 0.08–0.21), when exposed to *B. bassiana* (Fig. 2). A similar trend was observed when predators were exposed to *C. javanica* where *A. swirskii* had a higher proportion of prey consumed (0.40, CI: 0.29–0.54) in comparison to *N. californicus* (0.31, CI: 0.22–0.44) and *N. cucumeris* (0.14, CI: 0.09–0.23).

When exposed to azadirachtin + pyrethrin, *A. swirskii* consumed a higher proportion of consumed *S. dorsalis* (0.31, CI: 0.21–0.42) compared to *N. californicus* (0.13, CI: 0.08–0.21), and *N. cucumeris* (0.08, CI: 0.08–0.04). Similarly, in the capsicum oleoresin, garlic, and canola oil extracts treatment, *A. swirskii* had the highest proportion of consumed prey (0.31, CI: 0.21–0.43) compared to *N. californicus* (0.22, CI: 0.15–0.34), and *N. cucumeris* (0.15, CI: 0.09–0.24) (Fig. 2).

### Oviposition

Analysis of the oviposition rate revealed that *N. cucumeris* in the control treatment initially (24 h) exhibited a relatively high oviposition rate (0.52 eggs per day, CI: 0.18–1.56) compared to *N. californicus* and *A. swirskii*, which initially had lower oviposition rates (0.01 per day, CI: 0.007–0.3, and 0.07, CI: 0.02–0.22, respectively) (Table 5). However, the rate of oviposition of both predators increased over time (0.036 per hour, CI: 0.0203–0.0539, respectively). On leaf discs treated with insecticides, oviposition decreased to 0.017% (CI: 0.0038–0.0329) in *N. californicus* and 0.0013 (CI: 0.0133–0.0102) in *A. swirskii*. Nonetheless, there were no discernable differences in the rate of oviposition among predatory mite species in insecticide treatments (Table 5).

**Table 5.** Daily number of eggs laid by the predatory mites (oviposition rate) following exposure to different insecticide treatments. Comparisons are based on the preplanned contrasts

Contrast	Predatory mite oviposition rate		Difference in daily predatory mite oviposition rate (*Δ*μ)	*Δ*μ LCL	*Δ*μ UCL	UCL*LCL > 0
C1	0.286	0.184**(%)**	0.097	0.075	0.55	*
C2	0.218	0.108	0.105	−0.0271	0.41	
C3	0.165	0.259	−0.086	−0.398	0.17	
C4	0.027	0.032	−0.005	−0.485	−0.03	
C5	0.169	0.334	−0.154	−0.673	0.12	
C6	0.229	0.089	0.132	−0.058	0.81	

The “Predatory mite oviposition rate” column includes 2 subcolumns displaying the daily number of eggs laid averaged across the 3 predatory mite species for the 2 conditions being compared. The conditions are listed in the same order as described in Table 2. Differences in daily predatory mite oviposition rates (*Δ*μ) were calculated by subtracting the mean of condition 1 from condition 2 for each contrast. Positive values indicate higher daily oviposition for condition 1, while negative values indicate higher daily oviposition for condition 2. The “LCL” and “UCL” columns represent the lower and upper credible intervals of *Δ*μ, respectively. Asterisks (*) denote significant differences between the contrast comparisons based on whether the product of UCL and LCL overlap with zero.

## Discussion

Insecticides are key to managing *S. dorsalis* in strawberries, but they can harm predatory mites used for pest control. This study found that spinetoram significantly reduced the survival of *A. swirskii*, *N. cucumeris*, and *N. californicus*. Overall, *A. swirskii* and *N. californicus* were most affected, while *N. cucumeris* was the least affected. Fungal insecticides (*B. bassiana* and *C. javanica*) had the least impact on the survival or the predatory mites. Furthermore, the results of this study highlight substantial variability in response to insecticides among predatory mite species, not only within the same genus but also across the entire family.

To minimize the impact of insecticides on nontarget organisms, many companies in the insecticide industry are focusing on developing chemistries that have a lower impact on beneficial insect species (Sparks et al. 2021). This shift is partly driven by changes in government regulations, which now mandate that all new insecticides undergo testing on beneficial insects during their development (Leahy et al. 2014). Reduced-risk insecticides such as spinetoram and cyantraniliprole are expected to have a low impact on beneficial organisms such as bees (Besard et al. 2011, Kim et al. 2022). However, many studies have shown that these insecticides can negatively affect other beneficial organisms, such as predatory mites (Duso et al. 2008, Kim et al. 2018, Barroso et al. 2022).

Predatory mites are particularly vulnerable to insecticides due to the multiple routes of exposure, including direct contact, exposure to insecticide residuals, and the ingestion of prey that may harbor residual insecticides (Gentz et al. 2010). However, as this study demonstrates, different predatory mite species exhibit varying susceptibility to insecticides. This variability is as a result of differences in the kinetics and dynamics of toxicological processes among these predators (Feyereisen et al. 2015, Van Leeuwen and Dermauw 2016, Duso et al. 2020).

Research on the acaricidal effects of spinosyns against Acari has yielded some conflicting results depending on the Acari group being studied. In the Tetranychidae family, some studies report no acaricidal effects, while others demonstrate significant acaricidal activity of spinosyns. For example, Cowles (1998) found that spinosad had little to no activity against *T. urticae* when applied directly to the leaves of plants in a nursery setting. In contrast, van Leeuwen et al. (2005) reported that applying spinosad directly to the roots of tomatoes grown in rockwool (systemic application) and directly onto the leaves (contact application) provided excellent control of *T. urticae*. Wang et al. (2016) found that applications of spinetoram, an analog of spinosad, reduced the developmental time of *T. urticae* from egg to adult. Additionally, Wang et al. (2016) reported that the fecundity, intrinsic rate of increase, and net reproductive rate of *T. urticae* increased, leading to outbreaks of this pest.

In contrast, the effects of spinosyns on the Phytoseiidae family have generally been negative. The consensus indicates that spinosyns are harmful to predatory mites (Schmidt‐Jeffris et al. 2021), with most studies showing that spinetoram is more harmful than spinosad. For example, Kim et al. (2018) reported high mortality rates for *P. persimilis* (97.0%) and *A. swirskii* (90.7%) following exposure to spinetoram residues. Similarly, studies by Beers and Schmidt (2014, 2016), Shearer et al. (2016), Bergeron and Schmidt‐Jeffris (2023), Mills et al. (2015) and Döker et al. (2015) found comparable levels of adult mortality in *G. occidentalis* and *I. degenerans* when exposed to spinetoram. On the other hand, spinosad has been reported to have varying effects on the survival of adult phytosiide mites, with effects ranging from harmless to harmful (Fountain and Medd 2015). For example, Kim et al. (2018) reported that spinosad had a low effect on the survival of adults of *N. cucumeris*, while a meta-analysis by Schmidt‐Jeffris et al. (2021) showed that spinosad was highly toxic to larvae of many phytoseiid mites. This, therefore, shows that direct integration of spinosyns with phytoseiids used as biological control agents can reduce their efficacy, disrupting biological control.

Diamides have been reported to be harmful to predatory mites under laboratory and field conditions (Mills et al. 2015, Beers et al. 2016, Shearer et al. 2016, Bergeron and Schmidt‐Jeffris 2023). However, in this study, more than 50% of the predatory mites survived even after 72 h of exposure to cyantraniliprole. This suggests that while cyantraniliprole may be harmful to predatory mites initially, its harmfulness appears to decrease with prolonged exposure. This opens up the possibility of integrating cyantraniliprole into pest management strategies for *S. dorsalis* in strawberries by utilizing a temporal separation period of at least 72 h. By carefully timing the release of predatory mites after the application of cyantraniliprole, its impact on phytoseiid mites could be minimized. Additionally, establishing pesticide-free areas (predatory mite refuge sites) could provide a hiding place for predators to escape the adverse effects of diamides. This approach will further enhance the efficacy of predatory mites in the presence of this active ingredient (Duso et al. 2020).

In this study, azadirachtin + pyrethrin was found to be less harmful to *A. swirskii* compared to *N. cucumeris* and *N. californicus*, suggesting that this insecticide could be effectively combined with *A. swirskii* in pest management strategies. While azadirachtin has been reported to be selective and less harmful to certain predators (Castagnoli et al. 2002, Duarte et al. 2020), pyrethrin, an active ingredient in Azera, has been found to be harmful to predators (Duso et al. 2008). Although the exact mode of action of azadirachtin is still unclear (Sparks and Nauen 2015), this active ingredient has been reported to have acaricidal properties that could be harmful to some predators in this case *N. cucumeris* and *N. californicus* (Marčić and Međo, 2015, Thao and Thuy, 2023). Additionally, azadirachtin functions as an antifeedant, oviposition deterrent, metamorphosis inhibitor, and an effective insect repellent (Mordue (Luntz) and Nisbet 2000, Trumm and Dorn 2000, [Bibr CIT0032][Bibr CIT0001]). These combined effects could potentially be detrimental to predatory mites, particularly with prolonged exposure beyond 72 h.

When exposed to capsicum oleoresin, garlic, and canola oil extracts, there was a rapid decline in the survival of predatory mites especially beyond 72 h. Similar findings were reported when *Orius insidiosus* (Say) (Hemiptera: Anthocoridae) was exposed to capsicum oleoresin, garlic oil, and soybean oil extracts (Herrick and Cloyd 2017, Cloyd and Herrick 2018). According to the label information, capsicum oleoresin + garlic and canola oil extract is a product designed to repel insects (Gowan Company 2024). However, research has shown that most of the above components do not pose a direct threat to the majority of natural predators (Bostanian et al. 2005, Cloyd et al. 2009, Cloyd and Herrick 2018). This is probably because they are designed to repel insects from feeding on plants rather than predators feeding on prey. This suggests that these extracts could be effectively integrated into a management program involving *A. swirskii*, *N. cucumeris* and *N. californicus*. Capsicum oleoresin + garlic and canola oil extracts have been reported to be effective in suppressing *S. dorsalis* populations in strawberries (Lahiri et al. 2024). Therefore, combining these extracts with predatory mites could further enhance *S. dorsalis* management in strawberries, especially when pest densities become too high for predatory mites to control effectively. This approach could be particularly useful in Florida strawberry fields during February to March when (Rahmani et al. 2015) *S. dorsalis* populations rapidly increase.

Commercial formulations of entomopathogenic fungi such as *B. bassiana* and *C. javanica* have been successfully used as an alternative to the chemical for the management of many agricultural pests, including various phytophagous mite species such *Tetranychus evansi* Baker & Pritchard (Trombidiformes: Tetranychidae) (Wekesa et al. 2005), and *T. urticae* (Sáenz-de-Cabezón Irigaray et al. 2003). However, since predatory mites share many evolutionary similarities with phytophagous mites, entomopathogenic fungi can also be detrimental to these beneficial organisms. In this study, we observed that exposure of *N. californicus* and *A. swirskii* to *B. bassiana* for more than 72 h led to a drastic decline in their survival. *Beauveria bassiana* is one of the many toxigenic entomopathogenic fungi that produce mycotoxins, especially beauvericin. These mycotoxins cause significant cytotoxicity in cells and also induce oxidative stress, ultimately leading to the death of the host (Mallebrera et al. 2018). Secondly, *B. bassiana* conidia produce chitinase and Pr1–Pr2 proteases as part of the epicuticle penetration process (Kim et al. 2010) to accelerate conidia-host penetration, which can also affect predatory mites. Therefore, the secretion of toxins and cuticle degradation of the predators could explain the observed decline in rapid decline in survival especially after 72 h of exposure.

Different strains of *B. bassiana* have been reported to be infectious to many predatory mites. For example, 3 strains of *B. bassiana* (DEBI008, F, and J.B.) were reported to cause significant mortality to *A. swirskii* especially after 72 h (Seiedy et al. 2015). Other studies reported similar findings when *A. swirskii* and *N. californicus* were exposed to *B. bassiana* (Castagnoli et al. 2005, Numa Vergel et al. 2011, Midthassel et al. 2016). Additionally, *B. bassiana* has been reported to affect the survival of *P. persimilis* when the predator was exposed to topical treatments and dry residues (Duso et al. 2008, Pozzebon and Duso 2010, Numa Vergel et al. 2011). Nonetheless, we observed that *N. cucumeris* was the least affected predator when exposed to *B. bassiana*. Similar observations were made by Jacobson et al. (2001) when *B. bassiana* was used in conjunction with *N. cucumeris* under greenhouse and laboratory settings.

Avertedly, when predators were exposed to *C. javanica*, there was a rapid decline in their survival after 96 h of exposure. *Cordyceps javanica* has been shown to possess low toxicity to the predatory mite *N. cucumeris* (Chen et al. 2020), *N. californicus* (Castillo-Ramírez et al. 2020), and *A. swirskii* (Zhang et al. 2015). The decline in survival observed beyond 96 can be attributed to the reported low toxicity of *C. javanica* and the fact that these entomopathogenic fungi require a longer time to kill their host (Inglis et al. 2001, Shah and Pell 2003). This provides an opportunity of conducting concurrent applications of *C. javanica* and predatory mite releases. This strategy could be implemented at the start of the season (October to December), when *S. dorsalis* populations are low, allowing for the use of stronger chemistries later in the season as pest pressures increase.

The low predation by *N. cucumeris* observed in this study could be as a result of the quality of predators obtained from commercial suppliers. Variations in commercial rearing conditions, especially the nutritional history of the predators, can significantly impact their performance. However, these effects can be reversed in successive generations if the predators are provided with more than one food source. (Dicke et al. 1989, Lopez and Smith 2016, Vangansbeke et al. 2023). Additionally, the provision of a food source that is not nutritionally ideal for the predators (such as thrips) can lead to low predation rates (Eubanks and Denno 2000, Wimmer et al. 2008, Schmidt et al. 2012), which would also explain the low proportions of *S. dorsalis* consumed by the predators in this study. This further emphasizes the importance of providing generalist predators with alternative food sources such as pollen even when target prey is in abundance, as this approach has been shown to enhance their efficacy in controlling pests (Beltrà et al. 2017, Benson and Labbe 2021, Etienne et al. 2021).

Although *N. californicus* prefers feeding on spider mites in its natural habitat (McMurtry and Croft 2003, McMurtry et al. 2013), in this study, *N. californicus* consumed the highest proportion of *S. dorsalis* larvae. The ability of *N. californicus* to feed on thrips has been demonstrated (Rahmani et al. 2015). Additionally, the possibility of developing a strain of *N. californicus* capable of feeding on thrips has also been demonstrated to be possible (Castagnoli and Simoni 1999). Early exposure of *N. californicus* to *S. dorsalis* as a food source could have also facilitated the predation rate observed (Zhu et al. 2022) or that the quality of *N. californicus* received from the commercial insectary was better than that of *A. swirskii* and *N. cucumeris*.

However, it is crucial to recognize that the insecticides tested could have direct or indirect impacts on predation, which are not yet fully understood. The literature on the effects of some of the insecticides tested in this study on the feeding behavior of other predators suggests that these insecticides do not significantly impact feeding. For instance, exposure of *G. occidentalis* to cyantraniliprole had no impact on its predation capability (Schmidt-Jeffris and Beers 2017). Exposure of *Delphastus catalinae* (Horn) (Coleoptera: Coccinellidae) to *C. javanica* had no impact on its capability to feed on *Aleurothrixus trachoides* Back (Hemiptera: Aleyrodidae) (Avery et al. 2020). Similarly, when *Thalassa montezumae* Mulsant (Coleoptera: Coccinellidae) fed on eggs of *Phalacrococcus howertoni* Hodges and Hodgson (Hemiptera Coccidae) that had been sprayed with *C. javanica*, its predation capability was not affected (Barahona et al. 2018). Although *B. bassiana* has been shown to have minimal effect on *N. cucumeris* when released to suppress *F. occidentalis* (Jacobson et al. 2001). It can negatively affect other predatory mites in the *Neoseiulus* genus (Michereff-Filho et al. 2022). For instance, feeding *Neoseiulus barkeri* (Hughes) on *F. occidentalis* treated with *B. bassiana* led to reduced longevity and fecundity of the predatory mite (Wu et al. 2015). Another study reported observing *P. persimilis* avoiding leaves that had been treated with *B. bassiana* and exhibiting heightened grooming behavior and prolonged foraging, which directly impacted its predation (Zhang et al. 2021).

Oviposition in many phytoseiids mites is closely linked to prey consumption (Sabelis 1990) and the predator’s ability to digest prey (Janssen and Sabelis 1992). Thus, any factor that limits prey consumption, for example exposure to insecticides, indirectly impacts oviposition. Our findings indicate that the oviposition rates of the 3 predators did not vary when exposed to different types of insecticides. However, the impact of some tested insecticides on the oviposition of predatory mites is well documented in existing literature. For instance, azadirachtin was reported to cause a significant reduction in oviposition of *N. californicus* and *Phytoseiulus macropilis* (Banks) (Bernardi et al. 2013). Comparable outcomes were reported with exposure of *P. persimilis* to bean leaves treated with azadirachtin (Duso et al. 2008).

Broad-spectrum entomopathogenic fungi like *B. bassiana* have been shown to affect the oviposition of both phytophagous (Shi and Feng 2009) and predacious mites (Thoeming and Poehling 2006, Wu et al. 2015, 2018, Ullah and Lim 2017, Michereff-Filho et al. 2022). For example, in a laboratory study, *B. bassiana* was reported to cause a significant reduction in oviposition of *A. swirskii* (Midthassel et al. 2016), while another study reported similar findings when *Typhlodromalus aripo* De Leon (Mesostigmata: Phytoseiidae) was exposed to the entomopathogenic fungus *Neozygites tanajoae* (Agboton et al. 2013). Additionally, the fecundity of *P. persimilis* was reduced when the predator was exposed to *C. javani*ca. (Numa Vergel et al. 2011). Therefore, although entomopathogenic fungi have a lesser impact on the survival and feeding of predatory mites and can be directly integrated into a pest management program involving predators, their application could still affect predator oviposition, potentially reducing overall efficacy. To mitigate this, establishing oviposition sites in the form of pesticide-free zones could provide refuges where predators can safely lay their eggs.

In conclusion, findings from this study indicate that the insecticides used to manage *S. dorsalis* in strawberry production affect the survival and feeding of *N. cucumeris*, *N. californicus*, and *A. swirskii*. Among all the tested insecticides, spinetoram had the most significant impact on feeding and oviposition, suggesting an incompatibility between this active ingredient and predatory mites. Additionally, this research highlights that there might be a potential for integrating cyantraniliprole, azadirachtin + pyrethrin, capsicum, garlic, canola oil extracts, and *C. javanica* in an *S. dorsalis* IPM program that involves the use of predatory mites. However, additional research on the ideal time to release these predators after insecticide application needs to be fully studied. Proper timing of when to release predators following insecticide application can minimize the impact of these chemistries on predatory mites, allowing for efficient suppression of targeted pests. Nonetheless, the transgenerational effects of these insecticides on these predatory mites remain to be fully studied.
